# PSGL-1 Inhibits the Incorporation of SARS-CoV and SARS-CoV-2 Spike Glycoproteins into Pseudovirions and Impairs Pseudovirus Attachment and Infectivity

**DOI:** 10.3390/v13010046

**Published:** 2020-12-30

**Authors:** Sijia He, Abdul A. Waheed, Brian Hetrick, Deemah Dabbagh, Ivan V. Akhrymuk, Kylene Kehn-Hall, Eric O. Freed, Yuntao Wu

**Affiliations:** 1 National Center for Biodefense and Infectious Diseases, School of Systems Biology, George Mason University, Manassas, VA 20110, USA; she3@gmu.edu (S.H.); bhetrick@gmu.edu (B.H.); dabbagh.deemah@gmail.com (D.D.); 2 Virus-Cell Interaction Section, HIV Dynamics and Replication Program, Center for Cancer Research, National Cancer Institute-Frederick, Frederick, MD 21702, USA; abdul.waheed@nih.gov; 3 Department of Biomedical Sciences and Pathobiology, Virginia Polytechnic Institute and State University, Blacksburg, VA 24061, USA; Iakhrymu@vt.edu (I.V.A.); kkehnhall@vt.edu (K.K.-H.)

**Keywords:** coronavirus, cellular antiviral factor, pseudovirion

## Abstract

P-selectin glycoprotein ligand-1 (PSGL-1) is a cell surface glycoprotein that binds to P-, E-, and L-selectins to mediate the tethering and rolling of immune cells on the surface of the endothelium for cell migration into inflamed tissues. PSGL-1 has been identified as an interferon-γ (INF-γ)-regulated factor that restricts HIV-1 infectivity, and has recently been found to possess broad-spectrum antiviral activities. Here we report that the expression of PSGL-1 in virus-producing cells impairs the incorporation of SARS-CoV and SARS-CoV-2 spike (S) glycoproteins into pseudovirions and blocks pseudovirus attachment and infection of target cells. These findings suggest that PSGL-1 may potentially inhibit coronavirus replication in PSGL-1^+^ cells

## 1. Introduction

The ongoing coronavirus disease 2019 (COVID-19) is a global pandemic afflicting more than 63 million people in over 200 countries and territories, resulting in more than 1.47 million deaths as of 1 Decmber 2020. Although efforts to develop effective treatments and vaccines are moving forward rapidly, SARS-CoV-2, the virus that causes COVID-19, continues to spread. Understanding virus–host interactions is critical to developing both therapeutics and vaccines aimed at containing the pandemic. P-selectin glycoprotein ligand-1 (PSGL-1, also known as SELPLG or CD162) is a host protein recently identified to possess broad-spectrum antiviral activity [1]. PSGL-1 binds to the selectin family of proteins, P-, E-, and L-selectin [2], and mediates immune cell tethering and rolling on endothelium’s surface promote cell migration into inflamed tissues [3]. In the context of viral infection, PSGL-1 has been identified as an IFN-γ-regulated inhibitory factor involved in blocking the infectivity of HIV-1 [4], and other viruses [1], through the steric hindrance of particle attachment to target cells [1,5].

The coronavirus spike (S) glycoproteins play an essential role in viral entry by binding the cell-surface receptor on target cells and mediating the fusion between viral and cellular membranes during virus entry [6]. The S protein is also the target of neutralizing antibodies generated by the infected host. Because of its central role in virus infection and adaptive immunity, the S protein is a prime target for developing antiviral therapeutics and vaccines. In addition to the host immune response’s adaptive arm, viral infections trigger an innate immune response, largely induced by IFN, that sets up an antiviral state. Hundreds of IFN-stimulated genes (ISGs) are induced by viral infection [7]. While the role of some ISGs in blocking the replication of particular viruses is well established, the vast majority of ISGs have not been characterized. Because of the significance of host innate immunity in viral transmission and replication within and between hosts, there is an unmet need to understand these antiviral inhibitory factors in detail.

## 2. Materials and Methods

### 2.1. Virus Assembly

The SARS-CoV S and SARS-CoV-2 S expression vectors were kindly provided by Gary Whittaker and Nevan Krogan, respectively. Pseudoviruses were assembled by co-transfection with SARS-CoV S or -CoV-2 S expression vector (0.5 μg), pCMVΔR8.2 (7.5 μg), and pLKO.1-puro-TurboGFP (10 μg) or pLTR-Tat-IRES-Luc (10 μg), with either pCMV3-PSGL-1 (2 μg) or pCMV3-Empty vector (2 μg) as previously described [1]. Virus supernatants were collected at 48–84 h and concentrated by ultracentrifugation. SARS-CoV-2 virus-like particles (VLPs) were assembled in six-well plates by co-transfection of HEK293T cells with 0.5 μg each of SARS-CoV-2 expression vectors Puro1 SARS-CoV-2 M, pLVX-EF1a-nCoV2019-N-C-2xStrep-IRES-Puro, and pcDNA3.1(+)-P2A-eGFP-E-nCoV2019 (gifts from Nevan Krogan, University of California, San Francisco [8]), and pcDNA3.1 SARS-CoV-2 S (a gift from Thomas Gallagher, Loyola University, Chicago) in the presence of 0.2 μg of pCMV3-PSGL-1 or pCMV3-empty expression vectors using polyethyleneimine (PEI) transfection reagent. After 48 h of transfection, VLP-containing supernatants were filtered through a 0.45 μm membrane and VLPs were collected by ultracentrifugation over 20% sucrose. Hybrid alphavirus-SARS-CoV-2 particles were assembled as described in [9]. Briefly, HEK293T cells were co-transfected with 2 μg of each of the SARS-CoV-2 structural protein expression vectors (S, N, E, M) and 10 μg of the alphavirus luciferase reporter genome vector and 2 μg of a PSGL-1 expression plasmid or a control empty vector. Cells were cultured for 48 h, and then the particles were harvested from the supernatant, filtered through a 0.45 μm filter, and then purified by gradient centrifugation. 

### 2.2. Viral Infectivity Assay

Pseudovirus particles produced in the presence or absence of PSGL-1 were used to infect Vero E6 or Calu-3 cells (ATCC). Cells were pretreated with CoV-2 Pseudovirus Infection Enhancer (CoV-2 PIE) (a gift from Virongy LLC, Manassas, VA, USA) for 1 h at 37 °C, and then infected for 5 h. Infected cells were cultured for three days. Cells were lysed in Luciferase Assay Lysis Buffer and quantified by using GloMax Discover Microplate Reader (Promega). Hybrid alphavirus-SARS-CoV-2 particles produced in the presence or absence of PSGL-1 were used to infect HEK293T(ACE2/TMPRSS2) cells (a gift from Virongy LLC, Manassas, VA, USA) in 12-well plates. Cells were infected for 2 h at 37 °C, washed, and then cultured in fresh medium for 5 h and then analyzed for luciferase activity using GloMax Discover Microplate Reader (Promega, Madison, WI, USA). 

### 2.3. Virion Incorporation of SARS-CoV S Proteins and PSGL-1

HEK293T cells were co-transfected with the Env-defective HIV-1 molecular clone pNL4-3/KFS (1 μg) and vectors expressing the S protein of either SARS-CoV or SARS-CoV-2 (100 ng) in the presence of PSGL-1 expression vector or an empty control vector (200 ng). Virions were analyzed by SDS-PAGE and western blot using antibodies against SARS-CoV spike proteins (Genetex, Irvine, CA, USA), PSGL-1 (KPL-1 clone), or HIV-Ig to detect CA protein p24. Incorporation of PSGL-1 in SARS-CoV-2 VLPs was analyzed using co-transfected cell pellets and particles (concentrated by ultracentrifugation through 20% sucrose) that were solubilized in lysis buffer containing 50 mM Tris-HCl (pH 7.4), 150 mM sodium chloride, 1 mM EDTA, 0.5% Triton X-100, 10 mM iodoacetamide (Sigma-Aldrich, St. Louis, MO, USA), and protease inhibitor cocktail (Roche, Basel, Switzerland). Lysates were analyzed by SDS-PAGE and western blot using anti-Strep antibodies to detect Strep-tagged SARS-CoV-2 N, anti-SARS-CoV-2 S2 (for detection of precursor S0 and S2), and an anti-PSGL-1 antibody (KPL-1 clone) to detect PSGL-1.

### 2.4. Viral Attachment Assay

Virus particles were incubated with Vero E6 cells (pre-chilled at 4 °C for 1 h) at 4 °C for 2 h. The cells were then washed five times with cold PBS and then lysed in NuPAGE LDS Sample Buffer (Invitrogen, Carlsbad, CA, USA). Cell lysates were analyzed by SDS-PAGE and western blotting as described [1].

## 3. Results

**Figure 1:**
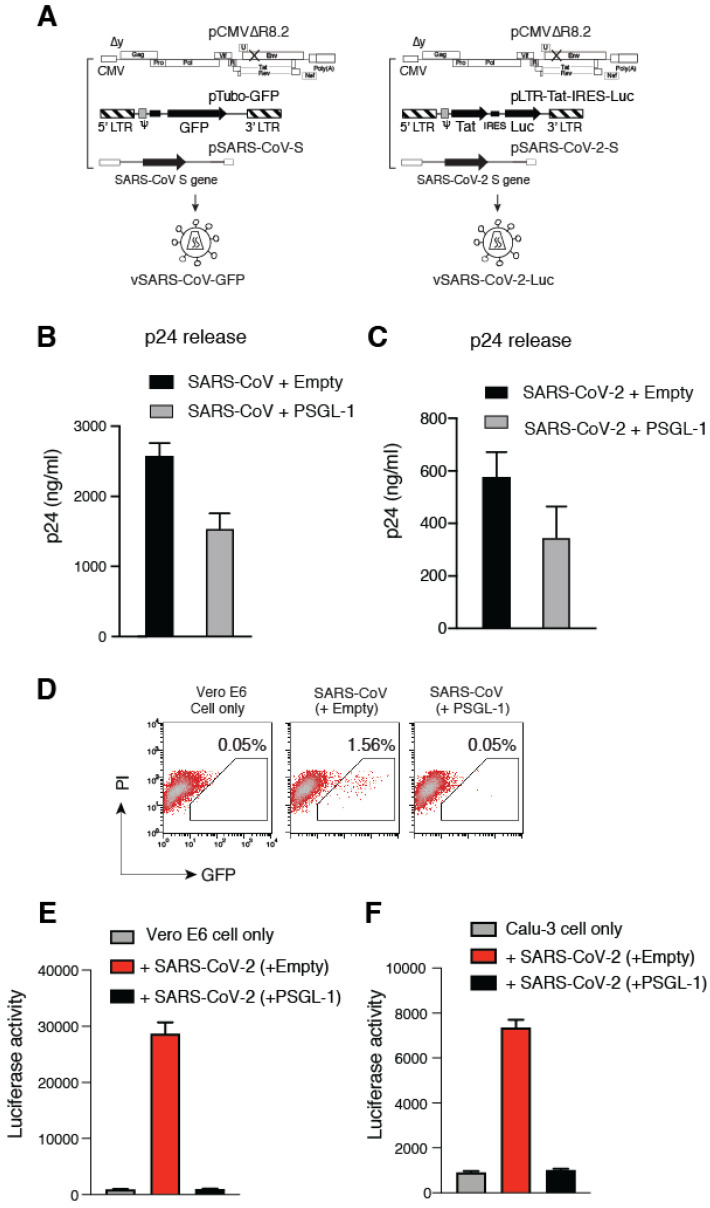
P-selectin glycoprotein ligand-1 (PSGL-1) inactivates the infectivity of SARS-CoV and SARS-CoV2 pseudoviruses. (**A**) Schematic of the assembly of lentiviral particles pseudotyped with the S proteins of SARS-CoV and SARS-CoV-2. (**B**,**C**) Effects of PSGL-1 on viral release. A PSGL-1 expression vector or control empty vector was co-transfected with the lentivirus packaging construct and a GFP or luciferase reporter plasmid, and viral release was quantified at 72 h post-transfection by HIV-1 p24 ELISA. (**D**) The infectivity of SARS-CoV pseudotyped virions was quantified by infecting Vero E6 cells and measuring GFP expression at 72 h post-infection. The percentages of GFP+ cells are shown. (**E**,**F**) The infectivity of the SARS-CoV-2 pseudotyped virions was quantified by infecting Vero E6 (**E**) and Calu-3 cells (**F**). Luciferase activity in infected cells was quantified at 72 h post-infection.

Previous studies have demonstrated that PSGL-1 can be incorporated into HIV-1 virions, and its virion incorporation subsequently blocks particle binding to target cells and infectivity [1,5]. To investigate the ability of PSGL-1 to restrict coronavirus infection, we first established a lentiviral vector-based coronavirus pseudovirus infection system, in which the S proteins from either SARS-CoV [10], or SARS-CoV-2 were used to pseudotype lentiviral particles (Figure 1A). Using this system, we assembled particles in the presence or absence of PSGL-1 [1], and then used the particles to infect target Vero and Calu-3 cells, which endogenously express the primary SARS-CoV and CoV-2 receptor, angiotensin-converting enzyme 2 (ACE2) [11,12]. The expression of PSGL-1 in viral producer cells had a minor (~ two-fold) effect on the release of SARS-CoV and –CoV-2 pseudovirions (Figure 1B,C), consistent with the previous finding that PSGL-1 expression has minimal effects on viral release [1]. However, the infectivity of PSGL-1-imprinted SARS-CoV particles was completely abrogated in Vero cells (Figure 1D), demonstrating the ability of PSGL-1 to block the infectivity of SARS-CoV S-bearing HIV-1 particles.

We further tested the effect of PSGL-1 on the infectivity of lentiviral particles pseudotyped with the SARS-CoV-2 S protein. We found that particles pseudotyped with SARS-CoV-2 S protein had much lower infectivity than those pseudotyped with SARS-CoV S protein. To resolve this technical issue, we developed a more sensitive reporter system in which a luciferase reporter (Luc) gene was expressed from the HIV-1 LTR in the presence of co-expressed HIV-1 Tat protein [13] (Figure 1A). A major advantage of this system is that high-level Luc expression can be achieved upon transactivation by co-expressed Tat protein following viral infection, minimizing non-specific Luc background from non-productive viral entry [14]. Using this system, we found that the infectivity of the SARS-CoV-2 pseudovirus is also potently inhibited by the expression of PSGL-1 in the virus-producer cells (Figure 1E and 1F). Together, these results demonstrate that PSGL-1 expression in the virus-producer cells severely diminishes the infectivity of virions bearing SARS coronavirus S proteins.

**Figure 2:**
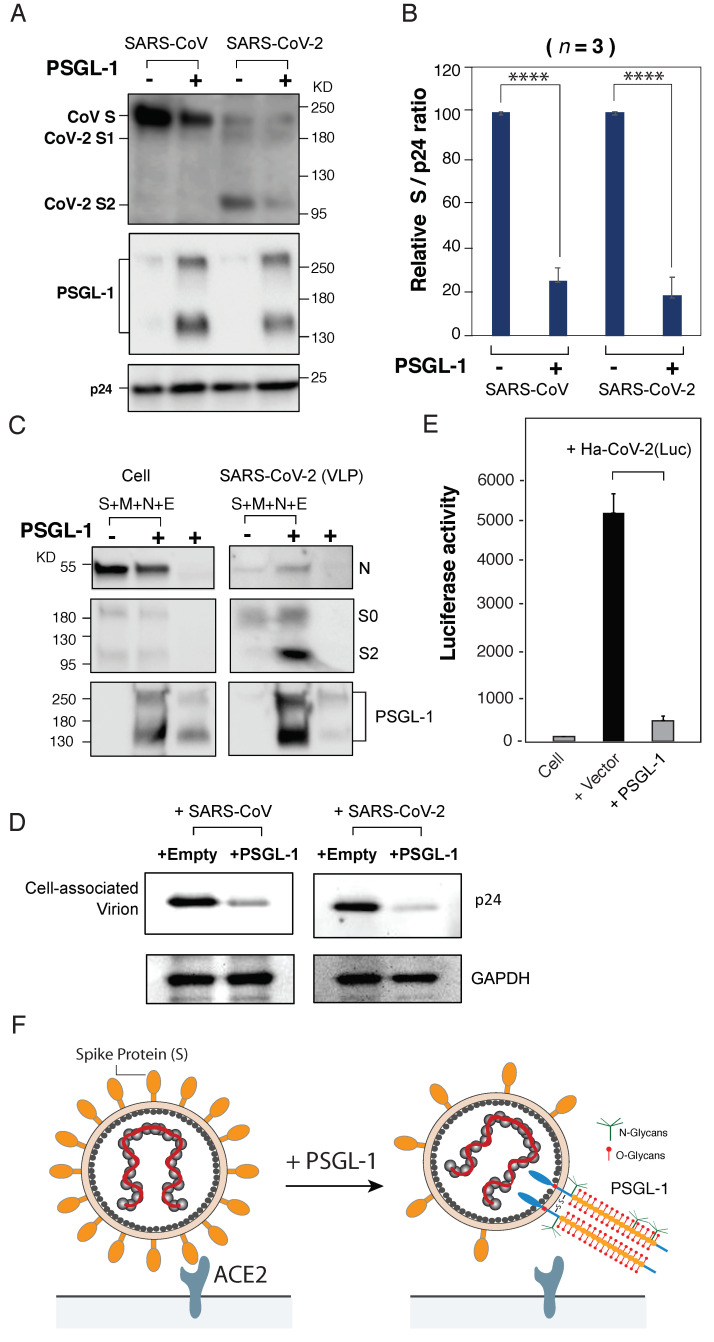
PSGL-1 inhibits the binding and entry of virus-like particles (VLPs) bearing SARS-CoV and SARS-CoV-2 S proteins. (**A**) PSGL-1 inhibits the incorporation of S proteins into lentiviral particles. Virions were produced from HEK293T cells co-transfected with HIV-1 pNL4-3/KFS DNA, the vector expressing either the SARS-CoV or the SARS-CoV-2 S protein in the presence of PSGL-1 or an empty control vector. Virion proteins were analyzed by western blotting using antibodies against SARS-CoV S proteins (Genetex), PSGL-1 (KPL-1 clone), or HIV-Ig to detect p24 (CA) protein. (**B**) The levels of SARS-CoV or SARS-CoV-2 S proteins in virions were quantified and normalized to viral p24 and set to 100% in the absence of PSGL-1. Data shown are + SD from three independent experiments. P values (two-tailed unpaired t-test): **** *p* < 0.0001. (**C**) PSGL-1 incorporation into SARS-CoV-2 VLPs. SARS-CoV-2 VLPs were produced from HEK293T cells by co-transfecting with vectors expressing SARS-CoV-2 M, N, E, and S proteins in the presence of PSGL-1 or an empty control vector. PSGL-1 expression alone in the absence of VLP production serves as a control to monitor the incorporation of PSGL-1 in exosomes. VLPs were pelleted and virion proteins were analyzed by western blotting using anti-Strep antibodies to detect Strep-tagged SARS-CoV-2 N, anti-SARS-CoV-2 S2 to detect precursor S0 and S2, and anti-PSGL-1 (KPL-1 clone) to detect PSGL-1. (**D**) PSGL-1 blocks virus attachment to target cells. An equal number of pseudovirions produced in the presence of a PSGL-1 vector or empty vector were assayed for attachment to target Vero E6 cells at 4 °C for 2 h. Cells were extensively washed, and cell-associated virions were analyzed by western blot for HIV-1 p24 (CA). GAPDH was used as a loading control. (**E**) PSGL-1 inactivates the infectivity of a hybrid alphavirus-SARS-CoV-2 reporter viral particle, Ha-CoV-2(Luc). Virion particles were assembled in the presence or absence of PSGL-1, and then used to infect HEK293T(ACE2/TMPRSS2) target cells. Luciferase expression was quantified at 5 h post-infection. (**F**) Model for the antiviral activity of PSGL-1 against SARS-CoV and CoV-2 S proteins. Left panel; in the absence of PSGL-1 in the virus-producer cell, virions bearing S protein bind the ACE2 receptor and infect the target cell. Right panel; expression of PSGL-1 in the virus-producer cell results in diminished S protein incorporation, and PSGL-1 incorporation into virions sterically blocks virus binding to target cells.

To investigate possible mechanisms, we analyzed the virion incorporation of SARS-CoV S proteins in the presence of PSGL-1. Our previous study showed that PSGL-1 could inhibit the incorporation of the HIV envelope glycoprotein [1]. As shown in Figure 2B, the expression of PSGL-1 in the virus-producer cell also decreased the amount of both SARS-CoV and SARS-CoV-2 S proteins on pseudovirions. To determine whether PSGL-1 is incorporated into SARS-CoV-2 VLPs, we assembled VLPs by co-transfecting HEK293T cells with vectors expressing SARS-CoV-2 S, M, E, and N proteins. Virions were harvested, partially purified through a 20% sucrose cushion, and then analyzed by western blot. We were able to detect PSGL-1 in SARS-CoV-2 VLPs (Figure 2C), demonstrating that PSGL-1 can be incorporated into SARS-CoV-2 particles during viral assembly. 

We and others previously reported that PSGL-1-mediated inhibition of virion infectivity is through the steric hindrance of particle attachment to target cells, which does not depend on the presence of viral envelope glycoproteins [1,5]. We performed a virion attachment assay and observed that the lentiviral particles pseudotyped with SARS-CoV or SARS-CoV-2 S protein produced from PSGL-1-expressing cells were impaired in their ability to attach to target cells (Figure 2D). These results demonstrate that the presence of PSGL-1 on virus particles can structurally hinder virion interaction with the target cells even in the presence of S proteins, consistent with previous studies of PSGL-1 and HIV-1 infection [1,5]. 

We next tested the ability of PSGL-1 to inactivate the infectivity of SARS-CoV-2 VLPs. For this purpose, we assembled a hybrid virion particle, Ha-CoV-2(Luc), that contains an alphavirus-derived Luc reporter genome inside SARS-CoV-2 VLPs [9]. Structurally, Ha-CoV-2(Luc) is composed of only SARS-CoV-2 structural proteins (S, M, N, and E), and requires both S and ACE2 for infection. Using the hybrid particles that were assembled in the presence or absence of PSGL-1, we were able to demonstrate that PSGL-1 expression in producer cells did not inhibit VLP production (Figure 2C); however, the infectivity of the Ha-CoV-2(Luc) particles was inhibited by expression of PSGL-1 in the VLP-producer cells (Figure 2E). Together, these results suggested the possibility that the presence of PSGL-1 in certain ACE2+ human cells may restrict SARS-CoV-2 replication in these cells.

## 4. Discussion

In this report, we demonstrate that the expression of PSGL-1 in virus-producer cells impairs the infectivity of virions bearing the S protein of either SARS-CoV or SARS-CoV-2, a phenotype shared among several other viruses (e.g., HIV-1, murine leukemia virus, and influenza virus) found to be sensitive to PSGL-1 restriction [1]. PSGL-1 has been suggested to be expressed in certain lung cancer cells [15], and in lung phagocytes that control the severity of pneumococcal dissemination from the lung to the bloodstream [16,17]. Nevertheless, it remains to be determined whether PSGL-1 is expressed in SARS coronavirus target cells in the lungs, and, if so, whether its expression can impair viral infection. Interestingly, recent studies suggested that SARS-CoV-2 may also enter and infect human T cells, monocytes, and macrophages, which express high levels of PSGL-1 [18,19]. Infection of T cells by SARS-CoVs could also potentially occur in the scenario of PSGL-1-mediated binding of T cells to inflamed human airway endothelium [20,21]. Nevertheless, it remains to be determined whether there is a role for PSGL-1 in blocking SARS-CoV-2 spreading infection in human cells that express PSGL-1.

In HIV-1 infection, the viral accessory proteins Vpu and Nef have been shown to antagonize PSGL-1 on CD4 T cells through surface downregulation and intracellular degradation [1,4]. It remains unknown whether coronaviruses possess a mechanism for antagonizing PSGL-1.

## 5. Patents

George Mason University has filed a provisional patent application pertaining to the results presented in this paper.

## Data Availability

All data generated or analyzed during this study are included in this article. Reagents are available from Y. W. upon request.
